# Comparison of Cell Viability and Chemical Composition of Six Latest Generation Orthodontic Wires

**DOI:** 10.1155/2021/8885290

**Published:** 2021-01-27

**Authors:** Lina M. Escobar, Jaime Rodrigo Rivera, Eliana Arbelaez, Luisa F. Torres, Andrea Villafañe, David Díaz-Báez, Ingrid Mora, Gloria I. Lafaurie, Midori Tanaka

**Affiliations:** ^1^Unidad de Manejo Integral de Malformaciones Craneofaciales UMIMC, Universidad El Bosque, Bogotá, Colombia; ^2^Posgrado de Ortodoncia, Facultad de Odontología, Universidad El Bosque, Bogotá, Colombia; ^3^Unidad de Investigaciones, Facultad de Odontología, Universidad El Bosque, Bogotá, Colombia; ^4^Unidad de Investigación Básica Oral UIBO, Universidad El Bosque, Bogotá, Colombia

## Abstract

Orthodontic wires are made of alloys containing different metals, including nickel. It is important to evaluate their biocompatibility prior to use, owing to their long-term use in patients. This *in vitro* study compared the cytotoxicity and chemical composition of six latest orthodontic wires: Fantasia®, Tanzo®, FLI®, NT3®, DuoForce®, and Gummetal®. The before-use group consisted of wires that were not used in the mouth, and the after-use group consisted of wires that were used in the mouth for two months. The wires were placed in contact with human gingival fibroblasts (HGF) for 72 h, and cytotoxicity was determined using the resazurin test. The chemical composition and surface characterisation were evaluated by spectrometry and scanning electron microscopy. The groups were compared using ANOVA and Kruskal–Wallis test. Only the FLI® wires produced a 36% reduction in HGF viability (*p* < 0.05) and presented greater irregularities and loss of polymer structure. After-use wires showed a significant reduction in the percentage of nickel and the appearance of new elements (oxygen and carbon). Therefore, it can be concluded that no toxic ion release was noticed in this study. Rhodium-coated wires were more stable than PTFE-coated wires, and only the FLI® wires showed a slight cytotoxic effect.

## 1. Introduction

Currently, several types of wires with various metal alloys are used in orthodontic treatment. Fixed orthodontic wires are made of stainless steel (SS) and nickel-titanium (NiTi), which consists of chromium, cobalt, nickel, and titanium [1]. The oral cavity has chemical and microbiological conditions conducive to the release of metal ions from alloys and coatings [2]. These conditions include the alkaline properties of saliva, electrolytes formed by food debris, variations in pH, mechanical action, and microbial flora [3]. Some of these released metallic elements can modulate the immune response causing inflammation [4], allergic and systemic reactions [5], and hypersensitivity [6]. Apart from allergic reactions, the release of ions may even cause cytotoxic effects, mutagenesis, and carcinogenesis [7]. There are great concerns regarding the biocompatibility of orthodontic wires and the ions related to their potential toxicity. It is a critical issue because of their long-term contact with the oral mucosa and the potential corrosion of different materials [8]. When choosing an orthodontic wire, it is important to have substantial knowledge of its composition, allergenic properties, and toxic effects. Additionally, a safety evaluation is required to consider the increasing variability of materials, their composition, and manufacturing processes since most orthodontists purchase materials that are commercially available without any concerns about their biocompatibility.

Among many alloys with shape memory materials, those with NiTi are the first choice of orthodontists because of their mechanical stability and biofunctionality [9]. Currently, there are several innovative Ni–Ti orthodontic arches available, offering orthodontists a variety of choices. Some of these new wires are superelastic nickel-titanium, nickel-titanium-copper, titanium-niobium, and nickel-titanium wires with rhodium or polytetrafluoroethylene coating.

The superelastic nickel-titanium wire is highly resistant to permanent deformation, presents consistent loading and unloading forces, and has a highly polished surface [10].

On the contrary, the new copper-nickel-titanium wires, according to the manufacturer, maintains consistent loading and unloading forces so that the arch provides the same predictable performance every time, allowing the arches to work in the mouth for longer intervals. They are highly polished, which reduces friction and the accumulation of debris [11]. The addition of copper to the nickel-titanium alloy improves the thermal properties of the wire while still allowing the force to be controlled. It is resistant to permanent deformation and temperature progression [11].

In copper-nickel-titanium thermoactivated orthodontic arch, copper provides less force than a nickel-titanium arch. It also has two different strength zones, making it possible to use arches of a rectangular section from the first phases of the treatment, thus performing faster three-dimensional control [12].

There is an increased demand for aesthetic components in orthodontics treatments; therefore, coated wires with an epoxy resin polytetrafluoroethylene (Teflon) or rhodium have been developed. These wires coated with rhodium have low reflectivity, which is promoted as being less visible and improving aesthetics, and the coating also resists peeling or cracking of the aesthetic layer [13]. The wires coated with polytetrafluoroethylene (PTFE or Teflon) have been found to be nonreactive, noncorrosive, and resistant to flaking. In addition, it has a low friction level during treatment [14].

Finally, the beta-titanium alloy combines a low-value elastic modulus with extremely high hardness. The elemental composition of this alloy is titanium-niobium-tantalum-zirconium [9]. Among its features are superelasticity, easy control of orthodontic strength, plastic deformation without any distortion, and low friction. In addition, because heavy metals do not contain heavy metals, they do not induce toxicity [9].

Since these wires have been recently commercialised, there is little evidence in the literature regarding their biocompatibility in the oral cavity and changes in their chemical composition due to their use in the mouth. Therefore, this study aimed to determine and compare the chemical composition of six latest generation orthodontic wires and evaluate their effect on the viability of human gingival fibroblasts *in vitro*.

## 2. Materials and Methods

### 2.1. Test Materials

This is an experimental *in vitro* and risk-free study, in which six recently marketed orthodontic wires were selected: Fantasia Wire® (International Orthodontics Services IOS, Stafford VA, USA), Tanzo® (American Orthodontics AO, Sheboygan WI, USA), FLI® (Rocky Mountain Orthodontics RMO, Denver CO, USA), NT3® (American Orthodontics AO, Sheboygan WI, USA), DuoForce® (Forestadent Pforzheim, Germany), and Gummetal® (Rocky Mountain Orthodontics RMO, Denver CO, USA) (Table 1). These wires were used during the first two months of orthodontic treatment, and once discarded, their chemical composition and cytotoxicity were analysed.

### 2.2. Collection and Storage of the Sample

Six different types of wires were collected from private clinics. For disinfection, the obtained wires were immersed in 0.5% enzymatic soap Bonzime (Laboratorios Eufar S.A, Bogotá Co.) for 5 min, washed with water, and kept in 2% glutaraldehyde (Sigma-Aldrich, Wuhan, China) for 3 h. Finally, they were rewashed with water, dried with paper towels, and stored in separate bags that were labelled with the type of alloy, size, number of samples, and date.

### 2.3. Cytotoxicity Test

Primary gingival fibroblasts, obtained from a human adult (HGF) (ATCC® PCS-201-018 ™), were used. The cells were seeded at 50000 cells/well in 24-well plates. They were maintained in Dulbecco's modified Eagle's medium (DMEM), supplemented with 10% foetal calf serum (FCS) and 1% penicillin/streptomycin and incubated at 37°C in a humidified 5% CO_2_ atmosphere for 72 h according to ISO 10993–5 standards [15], until they reached approximately 80% confluency.

The wires were divided into two groups. The before-use group included wires that had not been used in the mouth and the after-use group included wires that had been used for two months. In total, 90 wires were evaluated: 30 wires in the before-use group (5 cuts of each wire) and 60 wires in the after-use group (10 for each type of wire). Different numbers of wires were used in each group because before-group wires were understood as control specimens that had minimal variability in controlled laboratory conditions. As a negative control of cytotoxicity, cells without exposure to any wire were used, and as a positive control, HGF was treated with Triton X-100 (Sigma-Aldrich, St. Louis, MO). The wires were cut into 1-cm pieces and placed in contact with HGF for 72 h, as previously reported [7]. Subsequently, cytotoxicity was determined using the fluorometric resazurin test. After 72 h of placing the wires on the cells, the culture medium was extracted, and the wells were washed with phosphate-buffered saline (PBS). Subsequently, 400 *μ*L of DMEM medium without supplementation with 10% resazurin (v/v) starting from an initial solution of 44 *μ*M (Sigma-Aldrich, MO, USA) was added per well and incubated for 4 h under standard conditions to allow for the reduction of resazurin to resorufin [16]. At the end of the incubation period, the plates were read at excitation and emission wavelengths of 535 nm and 595 nm, respectively, in a microplate reader (Infinite 200 PRO Tecan Männedorf, Switzerland).

Since only metabolically active cells can cause this reduction, the amount of resorufin in each well was directly proportional to the number of viable cells present. Cell viability was then scored according to the classification of Ahrari et al. [17], as follows: more than 90% cell viability, no cytotoxicity (none); 60%–90% cell viability, slight cytotoxicity; 30%–59% cell viability, moderate cytotoxicity; and less than 30% cell viability, severe cytotoxicity.

### 2.4. Evaluation of the Chemical Composition and Surface Changes of the Wires

An analysis of the surface degradation of the wires was performed using scanning electron microscopy (SEM) equipped with energy dispersive spectroscopy (EDS) for the quantification of X-ray photons (JEOL - JSM-6490LV Peabody MA, USA). The changes in its chemical composition were determined through fluorescence spectrometry with EDS of each element present in the wires. Each section of all wires was analysed, and the final semiquantitative chemical composition corresponded to the average given by the chemical analysis software with correction type ZAF (*Z* = fluorescence), yielding percentage values of the atomic weight of each element present in the wires.

### 2.5. Statistical Analysis

The data was subjected to statistical interpretation by analysis of variance (ANOVA) for parametric data and the Kruskal–Wallis test for nonparametric data in the analysis of the comparison between groups. The normality of data distribution was tested using the Shapiro–Wilks test.

The Mann–Whitney test was used to determine which pairs of groups differed significantly from each other. The type 1 error rate was adjusted using Bonferroni correction. A comparison of the cytotoxic effect and chemical composition data for each type of wire in both groups (before and after-use) was performed using a paired *t*-test or Wilcoxon signed-rank test according to the distribution of the data. The analysis was carried out using the statistical package STATA (Statistical software: Release 14. College Station, StataCorp Cary, NC, USA). The data obtained by X-ray scattering spectroscopy (EDS) were analysed using the statistical program *R* (version 3.0.3, 2014, Chile). A *p* < 0.05 was considered statistically significant.

## 3. Results

### 3.1. Cytotoxic Effects of the Orthodontic Wires

**Figure 1:**
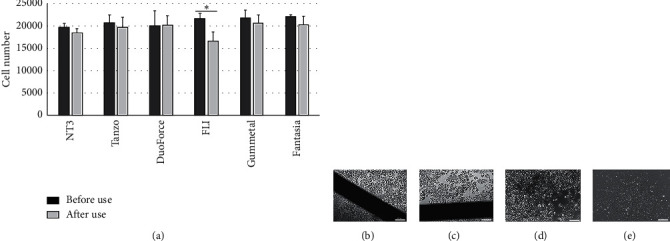
Cell viability (a). Number of fibroblasts obtained after cell culture with each type of wire, before or after use in the mouth. The FLI wire was the only one that produced a significant reduction in cell viability after use in the mouth (^*∗*^*p* ≤ 0.05). Photomicrographs of human gingival fibroblasts: HGF were observed in contact (72 h) with the NT3 wire before (b) and after use (c) in the mouth. Similar images were obtained with the other wires studied. The positive viability control corresponded to cells without contact with the wire (d), and the negative viability control was cells treated with Triton X-100 (e). Bar: 200 mm.

All the wires studied showed a minimal reduction in the viability percentages when the number of live cells was compared before and after use in the mouth. The percentages of cytotoxicity for NT3®, Tanzo®, DuoForce®, Gummetal®, and Fantasia® were 15%, 8%, 1%, 2%, and 12%, respectively. However, this reduction in viability was not statistically significant (*p* > 0.05). Only the FLI® wires showed a cytotoxic effect of 36%, which was statistically significant and corresponded to slight cytotoxicity (Figure 1 and Table 2).

### 3.2. Changes in Chemical Composition

**Figure 2:**
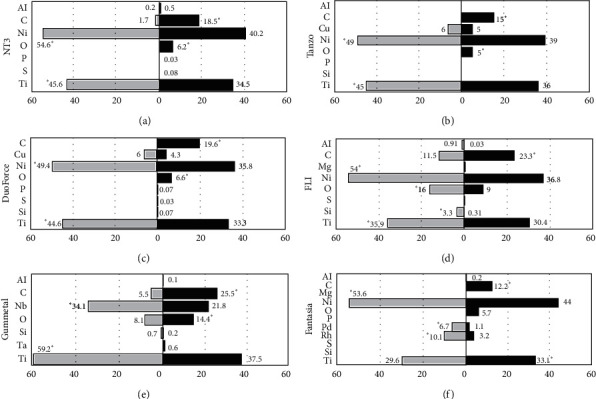
Chemical composition of six wires analysed: in the graphics, observed changes in the elemental composition (%) of NT3® (a), Tanzo® (b), DuoForce® (c), FLI® (d), Gummetal® (e), and Fantasia® wires (f) before and after their use in the mouth, ^*∗*^*p* ≤ 0.05.

When evaluating the changes in the chemical composition of the six wires, an increase in carbon (C) was found in NT3® wires, ranging from 1.7% in the before-use group to 18.5% in the after-use group. In the same group of wires, a reduction of titanium (Ti) and nickel (Ni) was observed, and 6.2% of oxygen (O) appeared in the after-use group (Figure 2(a)). Tanzo® wires also presented a reduction of 20% in Ni and Ti and an appearance of C (15%) and O (5%) in the after-use group (Figure 2(b)). In DuoForce® wires, Ni and Ti reduction was determined, similar to that found in the NT3® and Tanzo® wires. Elements C (19.6%) and O (6.6%) appeared while phosphorus (P), sulphur (S), and silicon (Si) were found in low proportions in the after-use group (Figure 2(c)).

On the contrary, FLI® arches showed an increase in C from 11.5% (before-use group) to 23.3% (after-use group). Additionally, a significant reduction was observed in Ni, Si, O, and Ti (Figure 2(d)).

Gummetal® arches presented a significant increase in C (from 5.5% to 25.5%) and O (from 8.1% to 14.4%), and reduction in niobium (Nb) and Ti in the after-use group (Figure 2(e)). In the Fantasia® arches, there was a reduction in Ni (44%), palladium (Pd) (1.1%), and rhodium (Rh) (3.2%). C and O appeared, and only Ti was increased to 33.1% in the after-use group (Figure 2(f)).

### 3.3. Wire Surface Alterations

**Figure 3:**
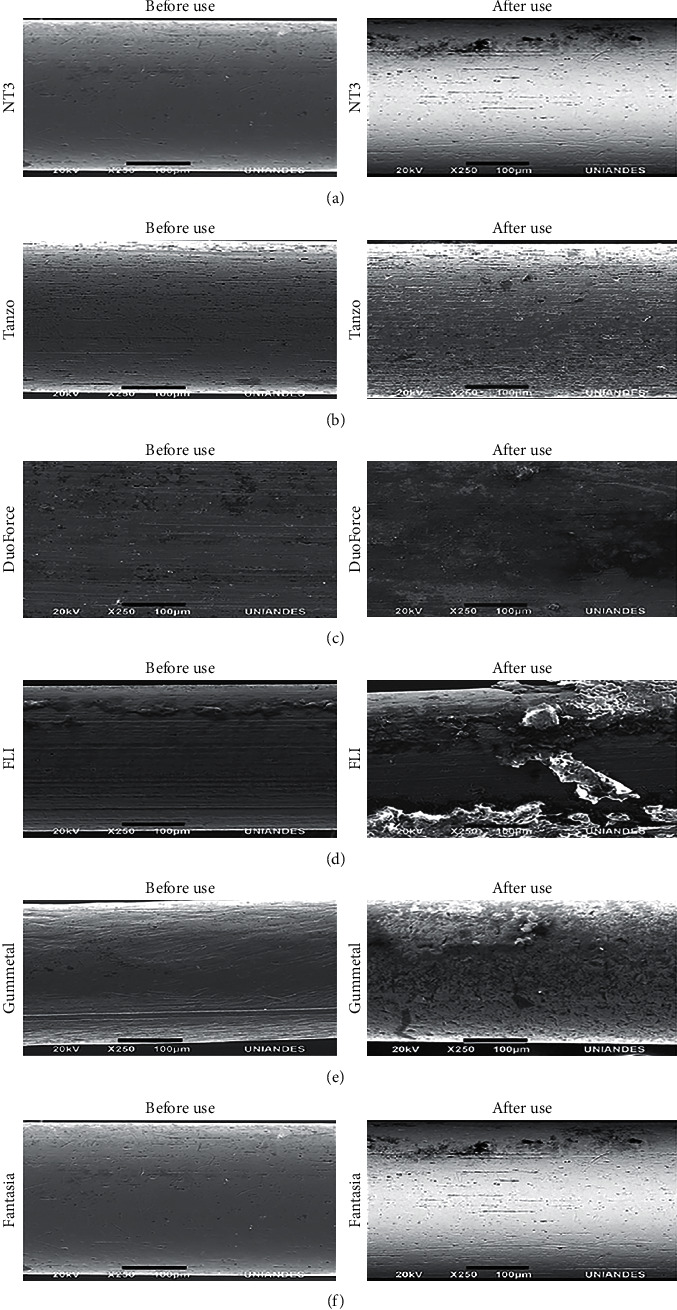
Scanning microscopy of wires examined: electron microscopy images of NT3® (a), Tanzo® (b), DuoForce® (c), FLI® (d), Gummetal® (e), and Fantasia® (f) wires before and after use in the mouth. Bar: 100 mm; original magnification: 250x.

Parallel lines, cracks, and wells were observed in the before-use group. These were deeper in the after-use group. Additionally, dark areas compatible with food debris or bacterial plaque were observed in NT3® (Figure 3(a)), Tanzo® (Figure 3(b)), and DuoForce® wires (Figure 3(c)).

In FLI® arches, a rough surface was observed that was compatible with its Teflon coating (Figure 3(d)). In the after-use group, flaking of this coating was observed. This resulted in a greater accumulation of substances such as plaque or food on its surface (Figure 3(d)).

In the group that used Gummetal® wires, a rough surface was observed owing to the absence of nickel. Additionally, nonparallel lines that increased in depth were observed in the after-use group. In the same group, dark areas, possibly due to plaque and food debris adhered to the wire, were observed (Figure 3(e)).

A rough surface was evident in the Fantasia® wires after-use group, with nonparallel lines, cracks, and wells that decreased in the before-use group (Figure 3(f)).

## 4. Discussion

The present study evaluated the biocompatibility of six elastic orthodontic archwires, two of which had aesthetic coating: FLI® wires coated with PTFE and Fantasia® wires coated with rhodium. A cell viability assay was used to evaluate the biocompatibility and cytotoxic behaviour of these new commercially available alloys, using HGF, since they are one of the main oral cells clinically exposed to the potentially toxic effects of orthodontic wires [18].

While performing the cell viability analysis, it was found that the wires without aesthetic coating did not display significant changes in the number of viable cells when the wires were compared before and after two months of use.

DuoForce® and Tanzo® wires presented similar cellular viability before and after the wire was used in the mouth. These results are similar to those found by David and Lobner et al. [19], who evaluated the cytotoxicity of nickel-titanium-copper wires on neuronal and glial cells from mice. Although nickel was present in these arches, they also contained titanium, which is known to decrease the release of metals due to the formation of a passive film, avoiding the generation of toxic effects in cells. The viability of fibroblasts exposed to Gummetal® wires was also not affected. Similar results were obtained by Niinomi et al. [20] while evaluating the cytotoxicity of beta-Ti–Nb–Ta–Zr wires on L929 cells derived from mice after 7 and 14 days. However, Rongo et al. [21] found slight cytotoxicity on days 1 and 7 but no cytotoxicity at 14 days when evaluating beta-titanium wires (TMA).

Additionally, two wires with aesthetic coating were evaluated: FLI®, a copper-nickel-titanium wire coated with PTFE, and rhodium-coated Fantasia® wires. On evaluating the cytotoxicity of rhodium-coated wires (Fantasia®), there were no induced changes in the viability of fibroblasts, as reported earlier [22].

Of the six wires evaluated, only the FLI® wire induced a statistically significant reduction in cell viability by 36%. The reduction in cell viability induced by FLI® wires corresponds to slight cytotoxicity according to the classification established by Ahrari et al. 2010 [17], and considering the ISO 10993–5 standard [23], for the biological evaluation of medical devices by direct contact, which states that when there is a reduction in cell viability of more than 30%, it is considered to have cytotoxic effects. Rongo et al. [21] determined the cytotoxic effect of several orthodontic wires with and without an aesthetic coating. The authors evaluated NiTi wires coated with Teflon Titanol Cosmetic® from Forestadent and found a slight reduction in the viability of HGF at all analysed times. These results are similar to those of this study, considering that FLI® wires also have a coating of PTFE, which progressively wears down with use in the mouth, generating a higher release of ions and a greater accumulation of plaque because of the surface irregularities, as previously reported [24]. Rongo et al. concluded that under experimental conditions, all the NiTi aesthetic archwires resulted in slight cytotoxicity, as did the uncoated wires. As such, their clinical use may have similar risks to uncoated archwires [21].

However, in the present work, the cytotoxic effect of uncoated nickel-titanium (NT3) wires was also evaluated for 72 h, without finding significant alterations in the number of cells during this time. These differences may be related to differences in the methodology and time of cytotoxicity evaluation, since, in Rongo et al., it was evaluated at 1, 7, 14, and 30 days. According to the previous results, aesthetic wires have mild cytotoxicity similar to metal wires, so their clinical use could be considered safe [21].

It is important to note that *in vitro* cytotoxicity tests do not completely represent the cytotoxic properties of materials in the oral environment. It is known that the oral mucosa is generally more resistant to toxic substances than cell cultures because of the presence of mucin and keratin layers [17]. However, cytotoxicity testing allows a comparison among available products and information for choosing a material with optimal characteristics.

Exposure time is another important parameter in cytotoxicity testing. The direct contact test between the wire and HGF resembles the actual exposure of the cells to the material as it would happen *in vivo*. However, the evaluation times reported in the literature are short, ranging from 24 to 72 h, as suggested by the ISO10993-5:2009 standard [7] for direct contact cytotoxicity studies. Few studies extend their analysis to more than 3 days [15, 21, 25] owing to the greater possibility of contamination, cell confluence, costs, and so on. Hence, in this work, a correlation between the cytotoxicity of the material and the evaluation time could not be established.

It is difficult to establish a direct correlation when there is *in vitro* cytotoxicity, but the use of the wire does cause acute clinical events *in vivo*. Therefore, the evaluation of subacute symptoms in clinical studies due to the use of this type of wire such as glossitis, metallic taste, bleeding, inflammation, or hypertrophied gingivae previously reported [26] for other materials should not be ruled out.

In this study, in addition to determining the cytotoxic effect, the changes in the chemical composition and surface alterations of the six titanium wires were also evaluated. Cytotoxicity induced by orthodontic appliances is related to the release of metal ions from corrosion processes. The release of substances from a biomaterial, whether from metal ions by corrosion of alloys or degradation of peroxides, can result in adverse effects such as toxicity, allergies, and mutagenicity. Additionally, exposure to ions could limit the recovery time necessary for cell repair [27, 28]. These findings are important as it has been found that degradation of the materials by electrochemical attacks, which are caused by factors such as temperature, quality, and quantity of saliva, bacterial plaque, pH, proteins, and chemical properties of solids and liquids in foods, can initiate the corrosion process and induce cytotoxic effects [29, 30]. Other studies have shown that a fluoridated and acidic environment such as that produced by creams or dental rinses increases the susceptibility to corrosion of certain metals, especially titanium [31, 32].

When analysing the chemical composition of the six wires studied, a reduction in the percentage of constituent elements after their use in the mouth was generally determined. The biodegradation of wire alloys can cause this reduction because the oral environment induces favourable ionic, thermal, and microbiological changes for the release of ions in the oral cavity [27]. Corrosion can roughen the appliance, increase the friction between archwires and slot, and release metal or alloy ions, which consequently can result in the discoloration of enamel and soft tissues, local pains, and allergic reactions in predisposed patients [33]. Ni and Cr are considered the most important elements among corrosion products owing to their ability to cause side effects. Corrosion resistance is among the basic principles of biocompatibility and depends on the type of alloy, manufacturing process, and surface features of the materials [34]. The results presented in this work show a significant reduction in the amount of Ni in the group of wires after its use in the mouth, except in the Gummetal wires that correspond to Ni-free alloys. Nickel is the most common metal that causes contact dermatitis and induces more cases of allergic reactions. The amount of nickel as the main constituent of contemporary orthodontic appliances may vary from 8% in stainless steel to more than 50% in NiTi alloys [35]. However, in most of the *in vivo* studies that have evaluated the liberation of metal ions from orthodontic appliances in biological fluids, it has been concluded that levels of metal ions do not reach the normal daily dietary intake of some elements [2]. Despite this, the possibility remains that even nontoxic concentrations of cations released from dental alloys might be sufficient to produce biological alterations (e.g., in DNA synthesis or alkaline phosphatase activity) [2].

Therefore, corrosion resistance is essential for orthodontic wires, not only because of the cytotoxicity and biological reactions that the released ions can generate but also because this can lead to the roughness of the surface, which severely limits the fatigue life and resistance to material breakage. Some alloys are resistant to corrosion because of their inherent nobility or the formation of a superficial protective layer [36].

Ti alloys depend on the formation of a passive surface oxide film to resist corrosion. However, even though these protective oxide films are present on the metal surface, metal ions can still be released. Not only is the protective oxide layer susceptible to both mechanical and chemical disruption, but the oxide film can also slowly dissolve as the wire is exposed to oxygen from the surrounding medium [37].

In this work, the appearance of new elements such as oxygen and carbon was observed in all wires, which could be related to the oxidation process of metals. The presence of these elements in the wires after use in the mouth can be caused by the adhesion of food debris, bacterial plaque, and the mechanism of passivation of the metal, where oxygen is taken from the environment as a protective mechanism [28].

When evaluating the morphological changes of the PTFE-coated wires, irregularities were found, and there was a loss of the polymer structure after 2 months of use in the mouth. These results are similar to those obtained in previous studies [38] that reported that the PTFE coating had poor stability because of the thinner coating layer of the as-received PTFE-coated aesthetic archwires than that reported by the manufacturer. Another study [39] also reported that PTFE-coated archwires showed the highest surface roughness after 28 days of immersion in artificial saliva at a pH of 6.75 when compared to epoxy resin.

In nickel-titanium NT3® wires, parallel lines, cracks, and wells were observed in the before-use group. These findings are similar to those reported by Puspitasari et al. [40], where the surface morphology of a superelastic nickel-titanium wire soaked in artificial saliva was found to have fibrous surfaces. In the NT3® wire group, after use in the mouth, we found dark areas compatible with food debris or bacterial plaque that may also be associated with pitting corrosion. This is similar to that reported by Puspitasari et al. [40] in a superelastic nickel-titanium wire that was soaked in artificial saliva. The nickel-titanium-copper wires, Tanzo® and DuoForce®, had similar characteristics to the NT3® wires, with a linear disposition in the arches before being used and higher roughness and indentations after use. In Fantasia® wires, nonparallel lines, cracks, and wells were observed after use in the mouth. Asiry et al. [22] compared epoxy, PTFE, and rhodium coatings and found that epoxy-coated wires had the highest surface roughness values followed by PTFE wires. Rhodium-coated wires were the best-coated wires in terms of surface roughness, comparable to those of uncoated wires. The SEM images showed remarkable changes in the PTFE coating layer on the wires after being used in the mouth. Additionally, the presence of cracks represented the deterioration of the external coating. Previous studies [38] reported that the PTFE coating had poor stability owing to the thinner coating layer. Moreover, this deterioration and coating loss exposes the core metal wire, causing absorption of large amounts of hydrogen because the titanium attracts hydrogen and undesirable aesthetic effects such as discoloration and ruptures that appear when the archwires are used clinically [37]. This results in a gradual change in the mechanical properties that must be considered.

To minimise biological risks, dentists should select alloys that have the highest biocompatibility and the lowest corrosion. The selection of an alloy should be made on a case-by-case basis using corrosion and biological data provided by the manufacturer or obtained in investigations of these new materials. A significant reduction in cell viability was only found in FLI® wires after being used in the mouth, with more evidence of superficial alterations owing to degradation of its coating. Although there were no critical changes in the chemical composition of the wires after being used in the mouth, there was a decrease in the level of all elements and the appearance of other elements, such as oxygen and carbon, possibly related to diet and metal passivation.

Many variables such as wear [41], brushing [42], and biomechanical stresses [43] can alter the metal release of orthodontic wires and the surface of dental materials. Therefore, further studies should examine these variables that cause these changes in wire structures and cytotoxicity, as can be an increasing concentration of metallic ions in the medium over time or a continuous release of substances as a result of the biodegradation of the aesthetic coating. Additionally, the study of factors involved in the loss of the aesthetic coating during wire use is needed.

Within the limitations of this report is the evaluation time of the cytotoxic effect on fibroblasts, which did not allow us to establish whether prolonged exposure of cells to different types of wires can increase cytotoxicity values. On the contrary, changes in the wire composition were determined after the use and deterioration of the aesthetic coating; however, studies must be carried out to establish what factors can cause these changes in wire structures and cytotoxicity. Additionally, clinical studies are necessary to evaluate the metal ion concentrations in patients undergoing orthodontic treatment with these types of archwires, considering variables such as wear, brushing, and biomechanical stress.

## 5. Conclusions

In conclusion, no toxic ion release was observed in this study. Of the six wires evaluated, only one that presented slight cytotoxicity was the FLI® wire. A significant reduction in the percentage of Ni was observed in all wires after their use, except in the Gummetal® wires. Elements C and O appeared on the wires after two months of use. Rhodium-coated wires were more stable after use than PTFE-coated wires. PTFE-coated wires showed greater irregularities and loss of polymer structure after two months of use in the mouth.

## Figures and Tables

**Table 1:** Archwires used in the study.

Orthodontic wires	Manufactured by	Alloy	Chemical composition
NT3	American orthodontics(AO®)	Nickel-titanium	45.6% Ti 54.6% Ni

Tanzo	American orthodontics (AO®)	Nickel-titanium-copper	45% Ti 49% Ni 6% Cu

DuoForce	Forestadent ®	Nickel-titanium-copper	44,6% Ti 49,4% Ni 6% Cu

FLI	Rocky mountain orthodontics (RMO®)	Polytetrafluoroethylene (Teflon) PTFE-coated copper-nickel-titanium	35,9% Ti 54% Ni 16% O 0,9% Al 3,3% Si 11,5% C

Gummetal	Rocky mountain orthodontics (RMO®)	Titanium-niobium	59,2% Ti 34,1% Nb 8,1% O

Fantasia	International orthodontics services (IOS®)	Rhodium-coated nickel-titanium	29% Ti 53,6% Ni 10% Rh 6,7% Pd

**Table 2:** Number of living-HGF cells exposed to different wires, before and after being used in the mouth, and percentage of cytotoxicity after 72 h.

Orthodontic wires	Before-use group	After-use group	Cytotoxicity (%)	*p* value
	Live cells mean ± SD			
NT3	19719 ± 852	18506 ± 891	15	NS
Tanzo	20838 ± 1633	19699 ± 2314	8	NS
DuoForce	20127 ± 3320	20242 ± 2051	1	NS
FLI	21771 ± 1016	16710 ± 1 932	36	*p* ≤ 0.05^*∗*^
Gummetal	21865 ± 1683	20734 ± 1686	2	NS
Fantasia	22132 ± 394	20379 ± 1796	12	NS

^*∗*^ *t-*test was used for statistical significance. NS: nonsignificant.

## Data Availability

The data used to support the findings of this study are available from the corresponding author upon request.
